# High-Output Cardiac Failure Revealing Primary Plasma Cell Leukemia

**DOI:** 10.1155/2013/504612

**Published:** 2013-03-04

**Authors:** Driss Chaoui, Bruno Gallet, Philippe Genet, Babette Mbungani, Ahmad Al Jijakli, Nina Arakelyan, Louisa Mesbah, Laurent Sutton

**Affiliations:** ^1^Service d'Hématologie, Hôpital Victor Dupouy, 69 rue du Lieutenant Colonel Prudhon, 95100 Argenteuil, France; ^2^Service de Cardiologie, Hôpital Victor Dupouy, 69 rue du Lieutenant Colonel Prudhon, 95100 Argenteuil, France

## Abstract

High-output cardiac failure in multiple myeloma (MM) is related to arteriovenous shunting in bone infiltrate disease. We describe such a complication in a patient with primary plasma cell leukemia (pPCL) without bone disease. We review the mechanisms that could be involved. As previously described, traditional cardiac failure therapy is not effective. pPCL therapy should not be delayed.

## 1. Introduction

Cardiac failure in plasma cell disease is mostly related to cardiac amyloidosis. High cardiac output may be associated with MM. A relatively high prevalence (23%) has been reported in multiple myeloma [1–3]. Several mechanisms have been described but arteriovenous shunting in bone infiltrate disease has the most supportive data [1, 4]. 

Primary or secondary PCL is a rare entity and a more aggressive disease than myeloma. Diagnosis was assessed when absolute circulating plasma cell was greater than 2 × 10^9^/L or greater than 20% of peripheral blood cells. In contrast to MM, extensive bone disease is uncommon in primary PCL (pPCL) [5] excluding in that case arteriovenous shunting as a mechanism of high cardiac index. 

We report and discuss the mechanisms of cardiac failure with high cardiac index in a patient with pPCL and without any bone involvement.

## 2. Case Report 

A 50-year-old man, without previous disease, was admitted to our hospital for dyspnea and epigastric pain. Heart rate was 130/min and blood pressure 110/60 mmHg. Both jugular veins were markedly turgescent and a gallop rhythm was found. Chest X-ray showed pleural effusion, which was exudative in laboratory examination and did not contain abnormal cells. Electrocardiogram showed sinus tachycardia without QRS, ST, and T changes. Spiral computerized tomography excluded pulmonary embolism. Echocardiography showed a high cardiac index: 12 L/min/m^2^, but no pericardial or ventricular dysfunction (LVEF 75%) and no argument for cardiac amyloidosis. Cardiac catheterisation findings were characterized by a high output cardiac state (12 L/min/m^2^). A diagnosis of high-output heart failure was assessed. 

Laboratory values were as follows: hemoglobin 9.8 g/dL, leucocyte count 18000/mm^3^, platelet count 68000/mm^3^, creatinine 136 *µ*mol/L, and calcium serum level 2.62 mmol/L; LDH level increased. Peripheral blood contained 35% of plasma cells. A monoclonal immunoglobulin G (IgG) kappa gammopathy (7.4 g/L) was detected. The percentage of plasma cells in bone marrow analysis was 19%. Chromosome 13 deletion was found on cytogenetic analysis. Immunophenotypic study revealed a CD38, CD138 positive staining, and CD19, CD20 and CD56 negative staining. No bone lesions were found in X ray studies of the skeleton. These results suggest the diagnosis of plasma cell leukemia.

Regular etiologies of high cardiac output such as thiamine deficiency, hyperthyroidism, and Paget's disease were excluded. 

The patient was first admitted to the cardiology unit. High doses of Furosemide were ineffective. A chemotherapy including VELCADE (Bortezomib) and dexamethasone was started. After one course of this treatment, no response was observed. Then, ALKERAN (Melphalan) 50 mg on day 1 in intravenous infusion was performed. Five days later, all circulating plasma cells were cleared. Cardiac failure also improved with rapid weight loss of 10 kg. No oxygen was needed and abdominal pain disappeared. Echocardiography performed 15 days later also evidenced an improvement of the cardiac index: 8 L/min/m^2^. A second course was performed at day 15. The patient left the hospital in complete hematological remission with no sign of cardiac failure. Three cycles of the classic Melphalan dexamethasone thalidomide were performed at 4-week intervals. 

Six months later, our patient experienced a relapse with circulating plasma cells. At the same time, right cardiac failure was assessed. A treatment with REVLIMID (lenalidomide) 25 mg daily dose without interruption was started. Plasma cells were cleared at day 30. Cardiac insufficiency signs also improved. Unfortunately, our patient relapsed one month later. REVLIMID was stopped. He died some days later of disease progression. 

## 3. Discussion

In the setting of plasma cell disease, arteriovenous shunting in bone lesions and humoral factors that affect cardiac function and peripheral vessel resistance are the two main explanations offered for high output cardiac failure. 

Regarding arteriovenous shunting, Sanchez et al. demonstrated that obliteration of abnormal pelvic vessel improved high output cardiac failure in myeloma patients with bone lesions [6]. The exact mechanism has been demonstrated by Inanir et al. in 11 myeloma patients with unexplained cardiac failure and high output. Arteriovenous shunting was found in all patients with a significant correlation with cardiac index and predominant shunt within the involved bone [4]. 

The second explanation is humoral factors. Kuribayashi et al. hypothesised that some unknown substance released by plasma cells dilated peripheral vessels, decreased systemic venous resistance, and enhanced the cardiac index. They found a high level of ammonemia and abnormal levels of amino acids such as glycine and tyrosine in 3 case reports. After chemotherapy, MM signs and cardiac failure improved. At the same time, serum level of amonemia and glycine decreased [7]. Other factors such as angiogenesis growth factors could be involved. Sasaki et al. have evaluated angiogenesis growth factors in an MM patient who experienced high-output cardiac failure. After chemotherapy, MM parameters and cardiac failure improved. Angiopoietin 2, insulin-like growth factor-binding protein 6, and glial cell line-derived neurotrophic factor increased before treatment and dramatically decreased after chemotherapy [8]. 

We favour the second hypothesis in our case. First, there is no evident of bone lesions. Second, the rapid correction of cardiac failure could not be due to the repair of the innumerable bone arteriovenous fistulas. Third, other etiologies such thiamine deficiency, hyperthyroidism, Paget's disease, and arteriovenous fistulae were discarded. Anemia was present at diagnosis but its correction did not improve cardiac failure. Moreover, McBride did not find a relationship between anemia and cardiac index [1].

Traditional heart failure therapies such as beta blockers, ACE inhibitors, and diuretics are not effective. The appropriate management is the correction of the mechanism involved, for example, embolization of abnormal communication in case of arteriovenous shunting [6] and the treatment of the underlying disease. The rapid efficacy of chemotherapy on PCL and cardiac failure supports this approach which should not be delayed. No response was observed with bortezomib which contrasts with previously published studies [9, 10]. A response was obtained with Melphalan but the patient rapidly relapsed, which did not allow intensive chemotherapy with ASCT. Angiogenesis is an important mechanism in the pathogenesis of plasma cell disease. We observed an improvement with lenalidomide but duration of response was short. We reported a slight improvement of OS in pPCL treated with new agents (Bortezomib, Lenalidomide, and Thalidomide) compared to previous studies before the use of novel agents [11, 12]. However, the benefit of these drugs seems to be less pronounced in PCL than in MM. The best approach has yet to be defined. In younger patients, Van de Monk et al. recommend Bortezomib-based regimen before and after ASCT with high dose Melphalan, followed by alloSCT or maintenance with novel agents. In patients ineligible for transplantation, induction and consolidation with Bortezomib followed by maintenance with novel agents were recommended [13]. 

In conclusion, high-output cardiac failure could be associated with PCL even in patients without bone lesions. Humoral factors are suspected to be the cause of this complication. Chemotherapy is the most appropriate treatment for both PCL and cardiac failure and should not be delayed.
